# Noninvasive vascular imaging of ruptured retinal arterial macroaneurysms by Doppler optical coherence tomography

**DOI:** 10.1186/s12886-015-0077-0

**Published:** 2015-07-22

**Authors:** Masahiro Miura, Daisuke Muramatsu, Young-Joo Hong, Yoshiaki Yasuno, Ayako Itami, Takuya Iwasaki, Hiroshi Goto

**Affiliations:** Department of Ophthalmology, Tokyo Medical University, Ibaraki Medical Center, 3-20-1 Chuo, Ami, Inashiki, Ibaraki 3000395 Japan; Department of Ophthalmology, Tokyo Medical University, 6-7-1 Nishi-Shinjuku, Shinjuku, Tokyo 1600023 Japan; Computational Optics Group, University of Tsukuba, 1-1-1 Tennodai, Tsukuba, Ibaraki 3058571 Japan

**Keywords:** Retinal macroaneurysm, Doppler, Optical coherence tomography, Indocyanine green angiography, Three dimensional, Optical coherence tomography angiography

## Abstract

**Background:**

To describe Doppler optical coherence tomography (OCT) imaging of ruptured retinal arterial macroaneurysms (RAMs).

**Methods:**

Four eyes of four patients with ruptured RAMs were prospectively studied. Vascular imaging was obtained using swept-source Doppler OCT, and compared with indocyanine green angiography images.

**Results:**

*En face* projection of Doppler OCT images clearly showed RAMs at the corresponding locations of lesions in the indocyanine green angiography images. In Doppler OCT images, RAMs were located in the inner retina in three eyes and in the medium layer of the retina in one eye. In one eye, detection of RAMs by standard OCT was difficult because of the presence of inner retinal hemorrhage. In one eye, disappearance of blood flow after direct laser photocoagulation could be confirmed by Doppler OCT images.

**Conclusions:**

Doppler OCT imaging may potentially function as a noninvasive complementary procedure with indocyanine green angiography.

## Background

Retinal arterial macroaneurysms (RAMs) are acquired aneurysmal dilatations of retinal arterioles, and usually develop within the first three orders of the arterial bifurcation [1, 2]. RAMs may remain asymptomatic and spontaneously resolve without treatment. However, rupture of RAMs causes preretinal and intraretinal hemorrhage, which may result in severe visual loss [1]. An accurate and rapid diagnosis is important for treating ruptured RAMs. In current clinical practice, the most reliable methods to detect RAMs are indocyanine green angiography (ICGA) and fluorescein angiography (FA) [1, 3]. However, the clinical applications of ICGA and FA have been limited because of patient discomfort and relatively long measurement times. Optical coherence tomography (OCT) has achieved micrometer-level axial resolution in cross-sectional retinal imaging, and has provided important information about the diagnosis and evaluation of therapeutic effects in RAMs [4–6]. However, standard OCT is only sensitive to backscattered light intensity and cannot provide information about blood flow. Because of this limitation, standard OCT has limited ability to detect RAMs.

Recently, a functional extension of OCT technology for three dimensional (3-D) vascular imaging was developed. This technique was first reported using Doppler OCT and was named optical coherence angiography [7]. Following this development, various 3-D vascular imaging techniques were reported, and were collectively called OCT angiography [8]. The clinical utilities of OCT angiography have been reported for polypoidal choroidal vasculopathy [9–12], choroidal neovascularization, [11–13] macular telangiectasia, [14] and diabetic retinopathy [15, 16]. Unlike standard OCT, OCT angiography can directly evaluate vascular architecture, including a comprehensive evaluation of vascular lesions in RAMs. In this paper, we evaluate the 3-D vascular architecture of RAMs using Doppler OCT angiography, and describe the clinical usefulness of Doppler OCT for RAMs.

## Methods

We prospectively evaluated four eyes of four Japanese patients with ruptured RAMs (one male, three females; age range, 67–92 years). The clinical characteristics of these patients are summarized in Table 1. The clinical diagnosis of ruptured RAMs was made by identifying macroaneurysms with ICGA. Eyes with severe cataracts or other eye diseases that interfered with Doppler OCT image quality were excluded from this study. One eye was treated with direct laser photocoagulation.

**Table 1:** Ruptured retinal macroaneurysm imaged by Doppler optical coherence tomography: Summary of subjects

Case	Gender	Age (years)	Eye	BCVA	Topographic location of retinal macroaneurysm	Associated findings
1	F	75	R	0.3	Medium layer of retina	Vitreous hemorrhage
2	M	73	R	0.6	Inner retina	Vitreous hemorrhage
3	F	92	R	0.05	Inner retina	Vitreous hemorrhage
4	F	67	R	0.9	Inner retina	

Visual acuity was measured on a Landolt chart. *M* male; *F* female; *R* right; *BCVA* best corrected visual acuity

The Doppler OCT system used in this study was a custom-made prototype built by the Computational Optic Group at the University of Tsukuba [11, 16]. This Doppler OCT was based on a swept-source technology, and operated at an axial scan speed of 100,000 A-scans/s, using a swept-source laser at a central wavelength of 1060 nm. The probing beam power was set at 1.85 mW, which is lower than the American National Standards Institute safety limit. The axial resolution in tissues was 6.4 μm. In a single scan, the system simultaneously provided both an intensity-based standard OCT image and a Doppler OCT image. The Doppler signal was calculated from two A-lines in two successive B-scans. Doppler signals were displayed in the form of the squared energy of the Doppler phase shift. Raster scanning protocols with 256 A-lines × 2048 B-scans covering a 6.0 × 6.0-mm region on the retina were used for volumetric scans. The acquisition speed of each measurement was 6.6 s/volume. Composite color Doppler OCT images, in which the Doppler OCT signal was overlaid onto the standard OCT with red color, were created from standard OCT and Doppler OCT images to specify the location of blood flow in the standard OCT image. *En face* vascular images were calculated from Doppler OCT volume data sets.

This study was performed according to the tenets of the Declaration of Helsinki, and was approved by the Institutional Review Boards of the University of Tsukuba and Tokyo Medical University. Informed consent for the study was obtained from all participants.

## Results

**Fig. 1:**
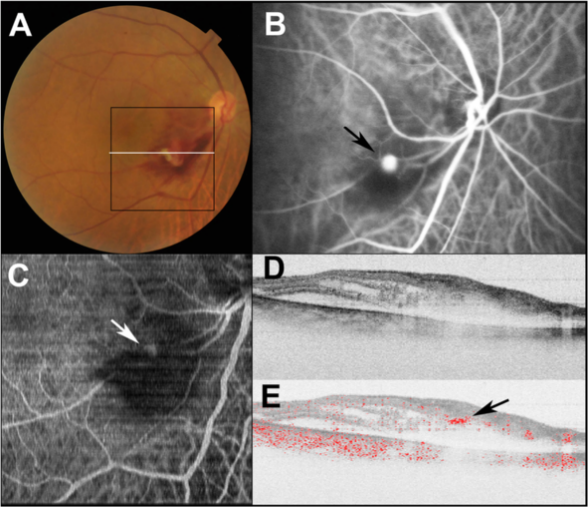
Doppler OCT images of the right eye of Subject 1 with ruptured retinal macroaneurysm. **a** Color fundus photograph shows retinal hemorrhage with a white lesion. Black line indicates scanning area of Doppler OCT (C), and white line specifies the B-scan OCT imaging (D, E). **b** Indocyanine green angiography image shows retinal macroaneurysm (black arrow). **c** An *en face* projection image by Doppler OCT shows retinal macroaneurysm at the corresponding locations of lesions in the indocyanine green angiography image (white arrow). **d** Standard OCT B-scan image shows inner retinal hemorrhage. **e** Doppler OCT B-scan image shows blood flow at the retinal macroaneurysm (black arrow)

**Fig. 2:**
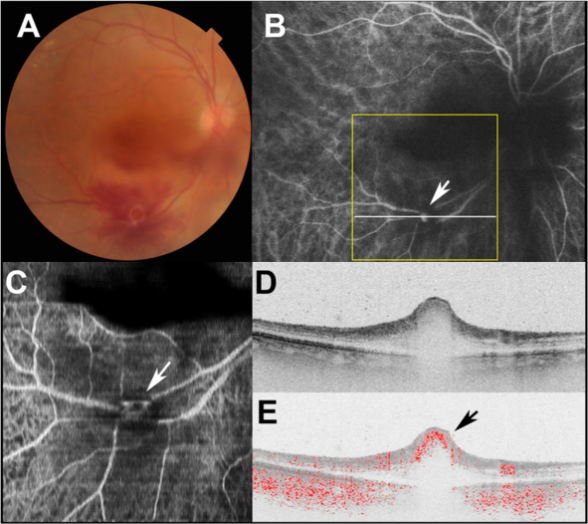
Doppler OCT images of the right eye of Subject 2 with ruptured retinal macroaneurysm. **a** Color fundus photograph shows retinal hemorrhage with a white lesion. **b** Indocyanine green angiography image shows the retinal macroaneurysm (black arrow). Yellow line indicates scanning area of Doppler OCT (C), and white line specifies the B-scan OCT imaging (D, E). **c** An *en face* projection image by Doppler OCT shows retinal macroaneurysms at the corresponding locations of lesions in the indocyanine green angiography image (white arrow). **d** Standard OCT B-scan image shows retinal macroaneurysm with focal bulge of the retinal surface. **e** Doppler OCT B-scan image shows blood flow in the retinal macroaneurysm (black arrow)

All eyes showed retinal hemorrhage around RAMs and three of four eyes were accompanied by vitreous hemorrhage. *En face* projections of Doppler OCT images clearly showed RAMs at the corresponding locations of lesions in the ICGA images (Figs. 1 and 2). Topographical locations of RAMs were readily determined by Doppler OCT B-scan images. RAMs were located in inner retinas in three eyes (Fig. 2). In these cases, RAMs could be readily detected with standard OCT B-scan images as focal protrusions of the retinal surface. In case 1, the RAM was located in the medium layer of the retina with the presence of inner retinal hemorrhage (Fig. 1). In this case, detection of the RAM in standard OCT B-scan images was difficult because of the absence of a focal change at the retinal surface of the RAM. In standard OCT B-scan images, active RAMs showed round high-intensity areas with low-intensity lumens in all cases.

**Fig. 3:**
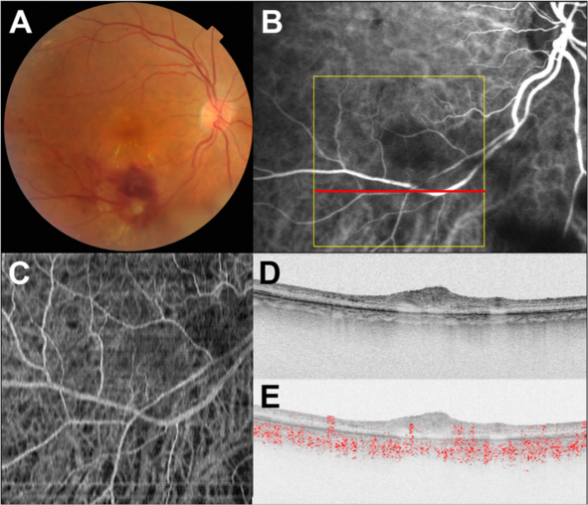
Doppler OCT images of the right eye of Subject 2 after direct laser photocoagulation at the retinal macroaneurysm. **a** Color fundus photograph shows decreasing retinal hemorrhage. **b** Indocyanine green angiography image shows disappearance of the retinal macroaneurysm. Yellow line indicates scanning area of Doppler OCT (C), and white line specifies the B-scan OCT imaging (D, E). **c** An *en face* projection image by Doppler OCT shows disappearance of retinal macroaneurysm as the indocyanine green angiography image (white arrow). **d** Standard OCT B-scan image shows high-intensity mass at the retinal macroaneurysm. **e** Doppler OCT B-scan image shows disappearance of blood flow at the retinal macroaneurysm (black arrow)

Case 2 underwent direct laser photocoagulation of the RAM. At 4 months after photocoagulation, ICGA showed occlusion of the RAM, and *en face* projection of Doppler OCT images clearly showed this occlusion. RAMs were filled with high-intensity areas in standard OCT B-scan images, and the disappearance of blood flow could be confirmed by Doppler OCT B-scan imaging (Fig. 3).

## Discussion

The clinical finding of RAMs varied greatly and was described as a masquerade-type syndrome [17]. Ruptured RAMs were accompanied by multilayer retinal hemorrhages, and precise diagnosis was difficult without ICGA or FA [1]. Doppler OCT imaging could noninvasively detect the RAMs without dye injection. The therapeutic effects of RAMs treatment could be readily detected with Doppler OCT imaging. This study shows the possibility of using Doppler OCT as an adjunct method to ICGA or FA.

In current clinical practice, the most reliable method to detect RAMs is ICGA or FA [1, 3]. However, neither ICGA nor FA could evaluate the topographic locations of RAMs because of their poor axial resolution [18]. Doppler OCT B-scan imaging could provide precise topographic locations of RAMs. In three of four cases, RAMs were located in the inner retina, as previously reported using commercialized spectral-domain OCT [4–6]. In these cases, detection of RAMs with standard OCT was relatively easy because of the presence of protrusions from the retinal surface at the RAMs. In one case, detection of RAMs by standard OCT imaging was made difficult by the lack of changes at the retinal surface. However, standard OCT findings for RAMs could be readily obtained using Doppler OCT images. After direct laser photocoagulation, disappearance of blood flow could be confirmed by Doppler OCT B-scan imaging, and lesions at the RAM were filled with high-intensity area images, indicating thrombus formation at the RAM. The combination Doppler OCT and standard OCT could therefore be useful for clinical evaluations of RAMs.

This study had several limitations. With the small number of subjects in our case series, we evaluated only some of the variations in RAMs. In this study, 6.6 s were required for a single measurement, despite using high-speed 100 kHz OCT. This relatively long measurement time caused motion artifacts in vascular imaging. Clinical applications of ultra-high-speed OCT have already been reported, indicating that the influence of motion artifacts could be minimized by shortening measurement times [19, 20].

This study demonstrated the clinical utility of Doppler OCT to evaluate RAMs. Doppler OCT could detect only some parts of the retinal vasculature; hence, ICGA or FA may still be required to more thoroughly evaluate the entire structure of vascular lesions. Doppler OCT could not detect dye leakage in FA and dye leakage was an important indicator of the activity of RAMs [1, 2]. However, the clinical applications of ICGA and FA have been limited by the possibility of adverse side effects [21]. Doppler OCT imaging is noninvasive, has a short measurement time, and may potentially function as a noninvasive adjunct tool to FA and ICGA for the assessment of RAMs.

## Conclusions

The swept-source Doppler OCT was used to evaluate four eyes of four patients with ruptured RAMs. Doppler OCT images clearly delineate the RAMs, and detect the therapeutic effect after direct laser photocoagulation. Doppler OCT imaging could provide important information for diagnosis and treatment of RAMs.

## Abbreviations

RAM: retinal arterial macroaneurysm
ICGA: indocyanine green angiography
FA: fluorescein angiography
OCT: optical coherence tomography
3-D: three dimensional
